# Edge effects and beta diversity in ground and canopy beetle communities of fragmented subtropical forest

**DOI:** 10.1371/journal.pone.0193369

**Published:** 2018-03-01

**Authors:** Marisa J. Stone, Carla P. Catterall, Nigel E. Stork

**Affiliations:** Environmental Futures Research Institute, Griffith School of Environment, Griffith University, Nathan, Qld Australia; AUSTRALIA

## Abstract

Clearing of dry forests globally creates edges between remnant forest and open anthropogenic habitats. We used flight intercept traps to evaluate how forest beetle communities are influenced by distance from such edges, together with vertical height, spatial location, and local vegetation structure, in an urbanising region (Brisbane, Australia). Species composition (but not total abundance or richness) differed greatly between ground and canopy. Species composition also varied strongly among sites at both ground and canopy levels, but almost all other significant effects occurred only at ground level, where: species richness declined from edge to interior; composition differed between positions near edges (<10 m) and interiors (> 50 m); high local canopy cover was associated with greater total abundance and richness and differing composition; and greater distances to the city centre were associated with increased total abundances and altered composition. Analyses of individual indicator species associated with this variation enabled further biological interpretations. A global literature synthesis showed that most spatially well-replicated studies of edge effects on ground-level beetles within forest fragments have likewise found that positions within tens of metres from edges with open anthropogenic habitats had increased species richness and different compositions from forest interior sites, with fewer effects on abundance. Accordingly, negative edge effects will not prevent relatively small compact fragments (if >10–20 ha) from supporting forest-like beetle communities, although indirect consequences of habitat degradation remain a threat. Retention of multiple spatially scattered forest areas will also be important in conserving forest-dependent beetles, given high levels of between-site diversity.

## Introduction

Globally, habitat destruction is a major contributor to biodiversity loss [1]. Forest cover has been reduced and fragmented by clearing, which has greatly increased the area covered by new forms of open anthropogenic habitat such as agriculture, pasture and suburbs. Patches of remnant forest then become bordered by high-contrast edges adjacent to surrounding open (matrix) areas, and forest microhabitats near these edges experience altered physical conditions, including increased wind disturbance and an associated risk of desiccation [2]. Concurrently, the abundances of individual species may change within the edge-affected zone, leading to altered community composition, diversity, and interspecific relationships [2–8].

Since Murcia’s [5] foundational review of edge effects in fragmented forests, further studies have improved knowledge of how insect communities are affected by edge creation, including research into beetles, true bugs, millipedes, spiders, and ants [6, 7]. Conceptual frameworks have focused particularly on the conservation implications for edge-avoiding species, especially when high ratios of edge to core habitat area in small or linear forest patches cause large reductions in effective habitat area, and when edge effects penetrate deeply into forest [2, 8, 9]. In such cases, retention of large habitat patches will be a high conservation priority [8, 10], since the edge-avoiding species would be expected to disappear from an equivalent total forest area that was more widely distributed among smaller patches, leading to regional declines in insect diversity.

However, there are also many cases where the abundances of certain species, or community diversity, are higher near edges [5, 10–12], with different implications for conservation planning. Spatial variability in community composition [13] would also complicate decisions about the existence and importance of edge effects across multiple sites. Further, the specific influence of edge distance on insect communities may be modified by additional factors such as forest type, vertical stratum, matrix type and structural forest-matrix contrast [7, 11, 14, 15]. Responses may also differ among target taxa or measured community attributes, for example in cases where distance from the edge affects species composition but not richness [7]. Therefore it is important to empirically test the generality of edge responses across different animal taxa, community attributes, ecosystem types and environmental contexts.

Many studies of forest edge effects on insect communities have focused on either moist tropical forests with high canopy cover or dense-canopy temperate forests [16]. There has been relative neglect of the more open drier forests, which comprise the original vegetation of about 43% of global tropical and subtropical land, often occurring in level, fertile locations [17]. These forests consequently have both high biodiversity value and globally threatened status [18], having suffered high deforestation rates worldwide [19]. Hence there is a need for research into edge effects on insect communities in dry open forest fragments, to fill the knowledge gap for this significant habitat type.

Here we evaluate how beetle (Coleoptera) communities within dry subtropical forest are influenced by distance from edges with open anthropogenic habitat, together with the additional factors of vertical height, spatial location, and local vegetation structure, in an urbanising region. We ask the following questions.

1. Are there edge effects on abundance, species richness, or species composition, how deep into the forest do they extend, and do they vary between ground and canopy levels?
2. What is the relative strength of community response to forest edges when compared with spatial variation among sites in multiple forest remnants, and with local habitat variation?

We also synthesise the findings of previous studies worldwide into the responses of forest beetle community abundance, species richness, and species composition at edges between remnant forest and open anthropogenic habitats. Finally, we consider the implications for conservation management.

## Methods

### Design and study region

**Fig 1:**
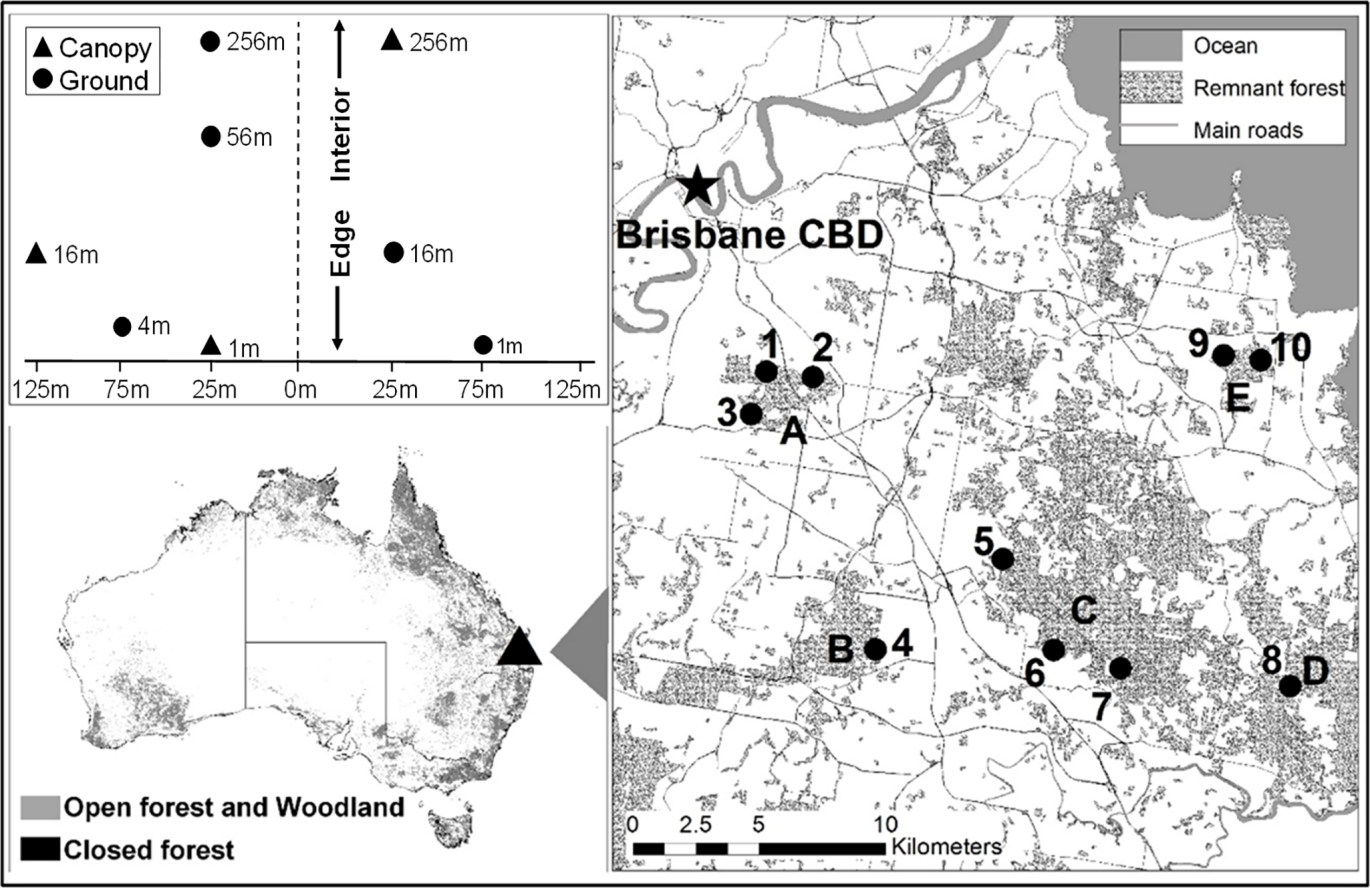
The study area and study sites. Sites are (numbered 1–10), in relation to major remnant forest areas which distinguish site-groups (letters A-E). Top left hand panel shows an example of the within-site spatial layout of traps; bottom left panel shows the study area’s location in relation to the distribution of forest in Australia [21].

We selected ten sites at forest edges where large (>200 ha) patches of subtropical open sclerophyll forest adjoined residential suburbs. The sites spanned an area of about 375 km^2^ in the southern greater Brisbane region of Queensland, Australia (27°31’32”- 27°39’25” S, 153°2’11”– 153°16’7” E; Fig 1). Sites were: at least 1.0 km apart; located at clearly defined edges; with 5–10 m cleared firebreak (or in one case a narrow road) between the forest and adjacent houses; 25–142 m asl and on ground that remained level up to 300 m from edges. Although standardising the orientation of edges may be desirable [3], our edges varied in aspect. The vegetation consisted of a tree stratum with multiple co-dominant species (mainly in the genera *Eucalyptus* and *Corymbia*), and an understory of grasses, shrubs and ferns, on moderately fertile shallow gravelly soils (Regional Ecosystem 12.11.5 [20]).

Within each site we sampled beetle communities along an an edge-to-interior transect consisting of five ground-level flight intercept traps at 1, 4, 16, 64 and 256 m from the edge, and three canopy traps at 1, 16 and 256 m (80 traps in total across the 10 sites). We staggered the traps at 1, 4 and 16 m laterally at randomly selected positions to achieve a minimum trap separation of 50 m (Fig 1).

The study region is mostly <200 m altitude, with average annual rainfall of 1000–1500 mm, mainly in summer, and average temperatures 9–21°C in winter and 18–27°C in summer (1949–2000 data [22]). Prior to European settlement in the mid-nineteenth century, the region’s dominant land cover was open eucalypt forest and woodland. Deforestation initially targeted the lowlands for agriculture followed by extensive suburban development from the mid-twentieth century. This left about 25–30% of the former forest uncleared by the late 1990s, with the larger remnants mostly confined to steep slopes associated with hills [20, 23].

### Beetle sampling and environmental measurements

The flight intercept traps (henceforth FIT) were deployed for four consecutive weeks during November—December 2014. Each FIT consisted of a clear polypropylene interception surface (40 cm by 50 cm) above a 5.0 l polypropylene container filled with detergent solution, similar to the design of Grimbacher et al. [13]. Canopy traps were suspended in the lower canopy (S1 Fig), at 6–16 m above ground (mean height 9.8 m, SE 0.53).

We mounted, labelled, and identified all sampled beetles to family and morphospecies (henceforth “species”) by MJS and NES, and cross-referenced them with beetle collections at Griffith University and Queensland Museum. Some subfamilies were also separately identified due to their distinctive biologies.

Each site’s distance to Brisbane’s central business district (CBD) was measured in kilometres. For some assessments of spatial effects, the 10 sites were grouped into five site-groups corresponding with different tracts of remnant forest (Fig 1). We measured eight local habitat variables at each ground-level trap location. Within a 1 x 1m^2^ plot randomly placed 2 m from each trap we visually assessed percent ground cover in five categories (litter, grass, twigs <1 cm diameter, bare ground and rock), and made one randomly placed measurement of litter depth (cm). Percent canopy cover was visually estimated directly above each trap. Intercept length (m) of coarse woody debris (diameter >10 cm) was measured along a 20 m line in the trap’s vicinity.

### Statistical analyses

We constructed sample-based accumulation curves and obtained total species richness (abundance coverage estimator; ACE) using EstimateS [24]. These were based on sample sizes of 10 sites, 8 traps /site (5 ground, 3 canopy), and 4 repeat collections/trap (total 320 samples). We estimated sample coverage using iNEXT v.1.0 [25]. Accumulation curves were separately constructed for ground and canopy at different edge distances, each based on N = 40 samples (10 sites x 4 collections), with 100 randomisations, and extrapolated to 80 samples. Non-overlapping confidence intervals at 84% (rather than 95%) were used to most reliably indicate significant differences, based on [26].

For all other analyses, we combined the four weekly collections from each trap to give one sample per trap. Following preliminary analyses which revealed non-linear relationships, we logarithmically transformed edge distance values (1, 4, 16, 64, 256 m) in statistical analyses. Unless stated otherwise, total abundance, species richness, and individual species’ abundances were log (x+1) transformed, to remove zero values, better fit parametric assumptions, and down-weight occasional large values from patchily abundant species.

We tested for an effect of edge distance on total abundance and species richness using 1-factor ANOVA, for ground (5 distances) and canopy (3 distances) separately (N = 10 replicate sites for each distance). These ANOVA’s were supplemented by linear regression of beetle variables on edge distance (N = 50 traps). Spatial differences in total abundance and species richness among the 10 sites were also tested using 1-way ANOVA with post hoc Tukey’s tests (N = 5 edge distances per site). If the site effect was significant, we standardised the relevant beetle variable by subtracting the site mean from each relevant trap value, and repeated the ANOVA test of edge distance.

We used Pearson’s correlations to test the effects on total abundance and species richness of: (1) the eight local habitat variables (canopy cover, woody debris, litter depth and five ground cover types) at ground level (N = 50 traps); and (2) trap height at canopy level (N = 30 traps). We also used Pearson’s correlations to test for relationships between edge distance (1, 4, 16, 64 and 256m) and the local habitat variables.

Patterns of variation in species composition among traps were visualised through a series of non-metric multidimensional scaling (NMDS) ordinations, using Bray-Curtis inter-site dissimilarities, with the ‘nmds’ function in ‘labdsv’ v1.8–7 ([27] in R [28] v3.2.2, with singletons excluded. We applied different sets of inclusion criteria for traps and species to various analyses, in order to display potential associations with trap height, and (for ground and canopy separately) with edge distance, site, and site-group. Edge distance ordinations used only “edge” and “interior” traps (defined respectively as <5 m and >50 m from the forest edge). These ordinations were repeated again except with the exclusion of certain species that were locally abundant at particular sites. To identify the species contributing most strongly to ordination patterns we used biplot vector overlays, through the ‘envfit’ function of ‘vegan’ v2.3.0 [29] in R. We also used biplot vector overlays to assess the strength and significance of association between relevant ordination plots and a range of environmental variables (edge distances, the eight local habitat variables listed above, and sites’ distances from the CBD).

To test for significant differences in species composition among edge distance categories, sites, and site-groups, we used Analyses of Similarity (ANOSIM) each with 9999 permutations in ‘vegan’ v2.3.0 [29], also in R. We further investigated the relative strength of association between environmental variables and the inter-trap Bray-Curtis dissimilarities using the distance-based linear model (DISTLM) tool in PERMANOVA+ [30]. We assessed edge-related variation in the amount of ground versus canopy difference in species composition (site-specific Bray-Curtis dissimilarity values) using one-way ANOVA among edge distances (1, 16, 256 m), with N = 10 replicate sites for each distance.

For ground and canopy separately, we assessed macro-spatial patterning in species composition at the site scale (N = 10 sites, with species’ abundances summed across edge distances within each site). We did this by comparing the inter-site Bray-Curtis dissimilarity matrix with a matrix of pairwise differences in sites’ distances from the CBD, using Mantel’s correlations in ‘vegan’, with 9999 permutations. We tested the ground-level correlation between site-specific edge-interior Bray-Curtis dissimilarity values (respectively based on species abundances averaged across pairs of edge-distances 1 and 4 m, and 56 and 256 m) and sites’ CBD distances. We also tested ground level correlation between site specific total abundances across pairs of edge-distances 1 and 4 m, and 56 and 256 m and sites’ CBD distances.

Where other analyses revealed statistically significant effects of environmental factors on species composition, we used the ‘indval’ function in ‘labdsv’ to further test for species whose abundances were most strongly affected. These involved effects of: height (all traps at ground versus canopy), edge distance category (ground level traps at 1 and 4 m versus 64 and 256 m; canopy traps at 1 m versus 256 m), canopy cover (17 traps with 50–85% cover and 18 traps with 15–30%) and site (10 sites). We excluded species occurring at <5 traps from trap-level analyses.

## Results

### Edge, height and site effects on the forest beetle communities

**Fig 2:**
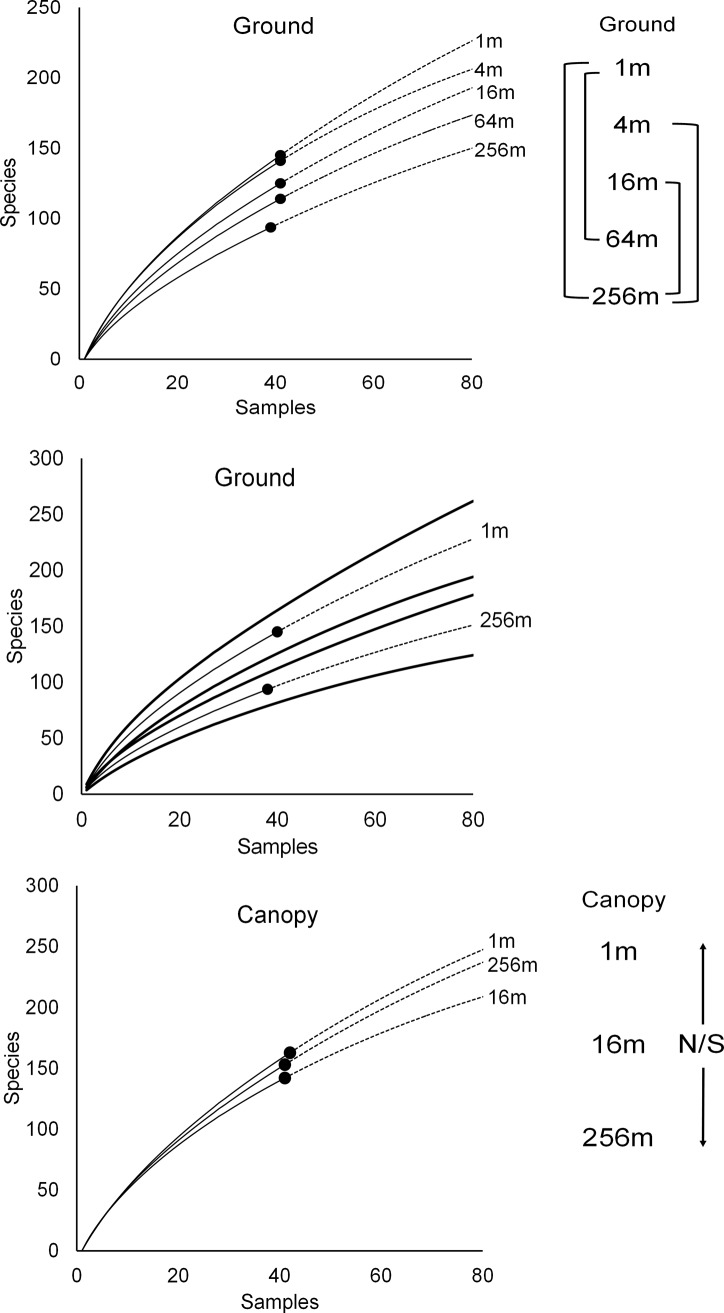
Sample-based species accumulation curves. The sample N = 40 trap-week X site combinations; with sampling endpoints shown by closed circles, then extrapolated (using dashed lines) to N = 80; significant differences are indicated by connecting lines in diagrams to the right. (top panel) Mean ground-level curves for each edge distance; (middle panel) 84% confidence intervals of significantly different curves at 1 m and 256 m from the edge; (bottom panel) mean canopy-level curves.

The sampling effort across all sites yielded 3,605 beetles of 578 morphospecies from 43 families, seven of which (Anobiidae, Chrysomelidae, Curculionidae, Histeridae, Scarabaeidae, Staphylinidae, Tenebrionidae) were further allocated to 16 specific ecologically-distinctive subfamilies, yielding 52 identified higher taxa at family/subfamily level, together with six “other subfamily” groups (S1 Table). The estimated total ACE species richness was 1,074 and sample coverage was 0.52, suggesting that about half of all potentially trappable beetle species were sampled. Accumulation curves for each edge distance showed a progressive decrease in ground level species richness from edge to interior, but no such edge effect at canopy level (Fig 2).

**Fig 3:**
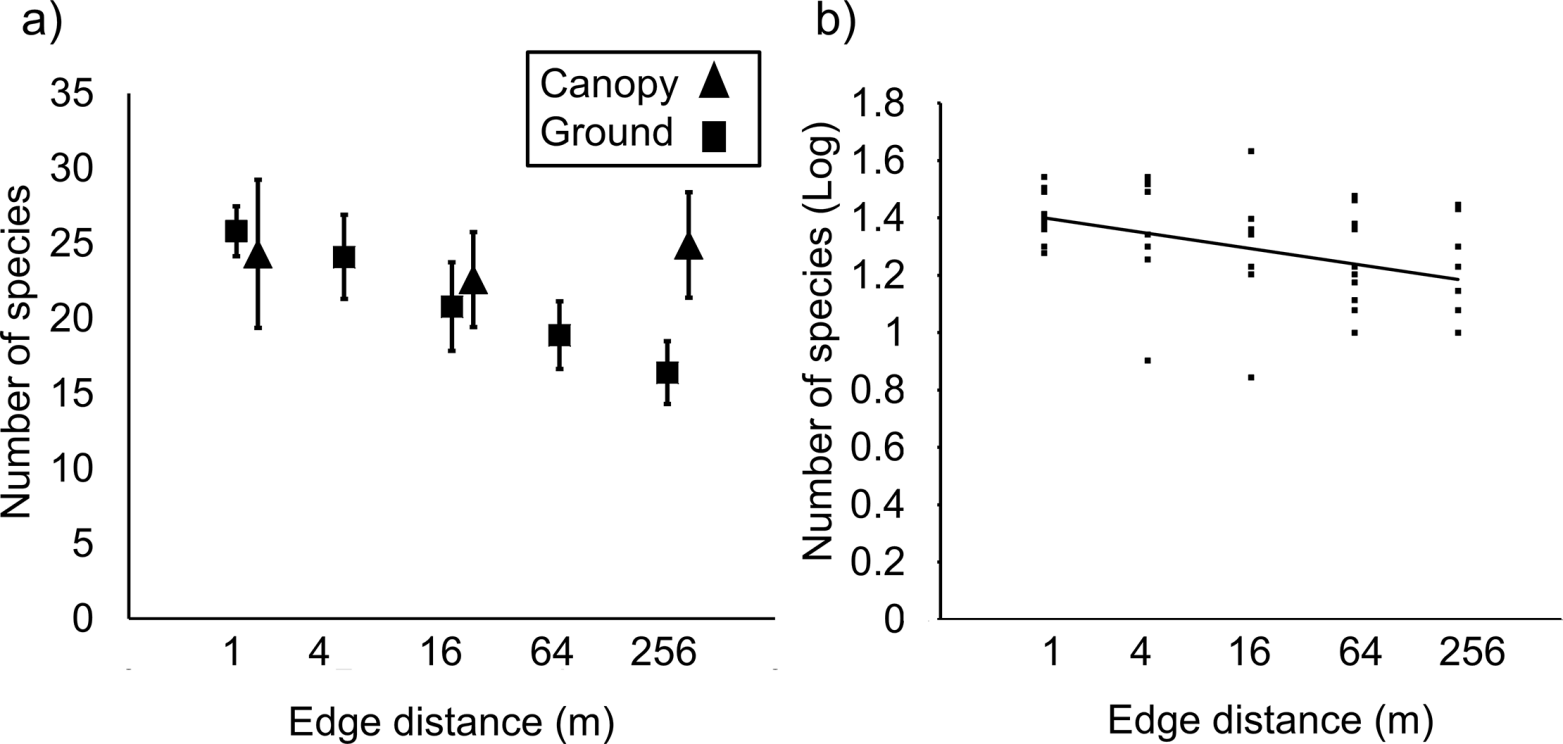
The effects of edge distance on beetle species richness. (a) Means and SEs at ground and canopy levels (each N = 10 sites); (b) logarithmic decline of ground-level species richness with increasing edge distance (N = 50 traps at different site-distance combinations).

The total number of beetle species per trap at ground level declined significantly with distance from edge in a logarithmic manner (Fig 3, Table 1), such that the greatest rate of richness decline per unit distance occurred within about the first 10 m from the edge. Species richness at canopy level was unaffected by edge distance (P = 0.73), and species richness did not differ among sites at either ground or canopy levels (Fig 3, Tables A and C in S2 Table; P values > 0.50). In contrast to the pattern for species richness, total beetle abundance was unaffected by edge distance (Tables A and C in S2 Table; P values for ground and canopy both >0.80), but differed significantly among both sites and site-groups, and was positively correlated (r = 0.40) with sites’ distances from the central business district (Table 1). Neither species richness (Fig 3) nor total abundance (S2 Fig) differed greatly between ground and canopy.

**Table 1:** Results for edge distance, other environmental variables, and spatial position.

Environmental factor	Ht^1^	Levels tested	Beetle attribute	Sig test^2^	Sample size	P
Height category	G&C	Ground, canopy	Composition	D	50, 29 traps	0.0001
Site	G	10 sites	Total abundance	A	5 traps/site	0.003
Site	G	10 sites	Composition	D	5 traps/site	0.0001
Site-group	G	5 site-groups	Composition	D	5–15 traps/gp	0.0001
CBD distance	G	numerical values	Total abundance	B	50 traps	0.004
CBD distance	G	numerical values	Composition	C	50 traps	0.0002
CBD distance	G	numerical values	Composition	F	10 sites	0.0004
Site	C	10 sites	Composition	D	9x3,1x2 traps	0.001
Edge distance	G	1,4,16,56,256 m	Species richness	A	5X10 traps	0.048
Edge distance	G	numerical values	Species richness	B	50 traps	0.006
Edge distance	G	numerical values	Composition	C	50 traps	0.04
Edge category	G	1&4, 56&256 m	Composition	D	2x20 traps^3^	0.05
Canopy cover	G	numerical values	Total abundance	B	50 traps	0.03
Canopy cover	G	numerical values	Species richness	B	50 traps	0.01
Canopy cover	G	numerical values	Composition	C	50 traps	0.01
Canopy cover	G	numerical values	Composition	E	50 traps	0.02
Canopy cover	C	numerical values	Composition	C	30 traps	0.02

Statistically significant effects of edge distance, other environmental variables, and spatial position on beetle community attributes (total abundance, sample species richness and species composition).

^1^ Height categories: G ground (traps at 5 edge distances), C canopy (traps at 1, 16, 256m); in each of 10 sites.

^2^ A ANOVA; B Pearson’s r; C extrinsic vector correlation with NMDS ordination; D ANOSIM; E DISTLM relationship with inter-trap Bray-Curtis dissimilarities; F Mantel test of environmental vs Bray-Curtis matrices. See Methods for details.

^3^ The result shown is based on 112 included species that were present at one or more traps, but excluding six locally-abundant site indicator species; excluding a further six less common indicator species gives P = 0.04, while including all 118 potential species gives P = 0.11)

**Fig 4:**
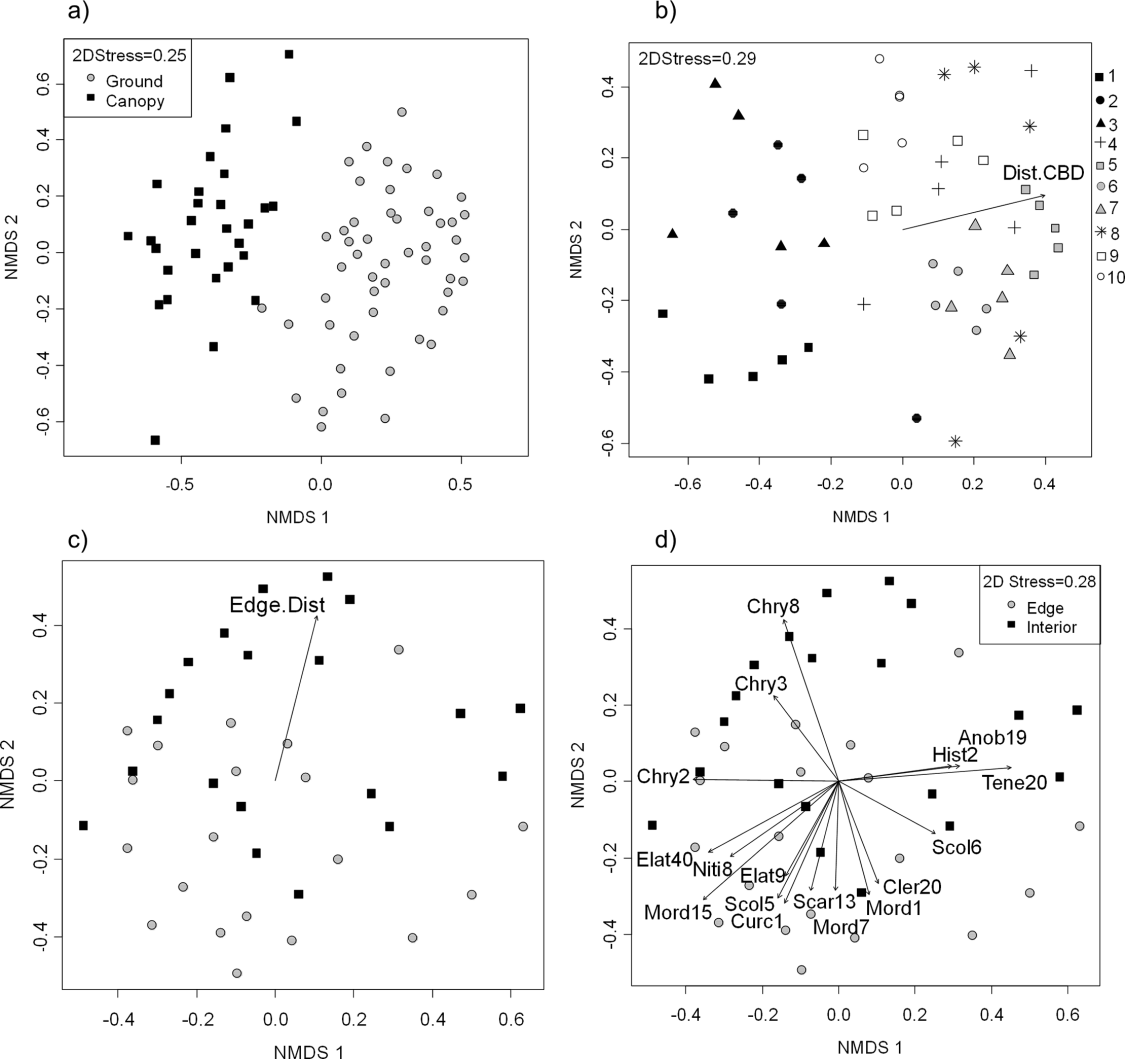
Beetle species composition NMDS ordinations. Patterns of variation in beetle species composition as revealed by NMDS ordinations, together with lines displaying biplot vectors for species or environmental factors that were significantly (P<0.05) associated with the ordination patterns. (a) Ground vs canopy differences (N = 50, 30 traps respectively, with 246 species). (b) Site-level variability at ground level (5 traps in each of 10 sites, with 142 species, Dist.CBD = distance to central business district). (c), (d) Edge-interior differences at ground level, using only traps at 1 and 4 m (edge) and 64 and 256 m (interior); each 20 traps, with 112 species (excluding six locally-abundant site indicator species); in (c) Edge.Dist = distance from forest edge; vectors in (d) show species.

Beetle species composition differed strongly between ground and canopy levels, and also differed strongly among sites at both ground and canopy levels (Fig 4, S3 Fig, Table 1). At ground level, the among-site variation in composition was also significantly correlated with sites’ distances from the central business district (Fig 4, Table 1), but this was not the case at canopy level (Table C in S2 Table; P = 0.15).

Variation in species composition at ground level was also significantly, but less strongly, associated with distance from the forest edge (Fig 4, Table 1). The community pattern at ground level was complicated by the influence of several common species which had disproportionately high abundance at certain sites (and contributed substantially to the compositional difference among sites), as indicated by both biplot vector overlays (Fig 4, S4 Fig) and IndVal analyses (Table B in S3 Table, S4 Table). Subsequent removal of these common species from the analysis clarified the edge effect’s statistical significance (Table B in S2 Table). Community composition at canopy level was not significantly associated with edge distance (P > 0.50, Tables B and C in S2 Table).

More individual species were strongly associated with the forest edge than with interior positions (respectively, <5 m and >50 m from the forest edge); as evident from both biplot vector overlays on the relevant ordination plot (Fig 4, S5 Fig) and IndVal analyses (Table B in S3 Table). These analyses identified a variety of different species with greater abundance at sites near the forest edge, whereas only one species (Chry8) was clearly associated with the interior.

The degree of compositional dissimilarity between ground and canopy traps within each site was not affected by distance from the forest edge: with respective mean Bray-Curtis values of 0.88, 0.79 and 0.88 at edge distances of 1, 16 and 256 m respectively (ANOVA F = 0.79, df = 2, 27, P = 0.46). The degree of compositional dissimilarity between edge (< 5m) and interior (>50m) positions within each site was not significantly associated with distance from the CBD (r = -0.44, P = 0.21, N = 10 sites).” The difference in total abundance (interior minus edge) was likewise not significantly associated with distance from the CBD (r = 0.17, P = 0.63, N = 10 sites).

### Effects of local habitat factors

Species richness and total abundance of beetles at ground level both had significant (though not strong) positive correlations with local canopy cover. But this was not the case for beetles in the canopy (Table 1; ground r values respectively 0.36, 0.30 (P = .03, 0.01); canopy P values both >0.90). Variation in species composition was also significantly associated with canopy cover, at both ground and canopy levels (Table 1), although only the former was supported by both DISTLM and Mantel analyses (Table C in S2 Table).

In turn, canopy cover had a significant negative logarithmic relationship with distance from the forest edge (r = -0.36, P = 0.009); canopy cover was about 50% within 5 m of the forest edge compared with about 35% at surveyed distances 16–256 m from the edge (Tables A and B in S5 Table). Together with their lower canopy cover values, positions further from the forest edge had significantly higher grass cover together with lower cover of twigs and bare ground (Table A in S5 Table; respective r values 0.41, -0,43, -0.31). However none of these or other local habitat attributes (woody debris, litter depth, percent cover of litter and rock at ground level, trap height at canopy level) were significantly associated with variation in species richness, total abundance or community composition (Table A in S5 Fig, Table C in S2 Table).

IndVal analyses of ground-level indicator species revealed four that were significantly or strongly (P<0.10) associated with denser canopies while one species was associated with sparser canopies; however, only one of these species (Elat38) was also listed among the six indicator species of edge or interior, being disproportionately abundant near the edge at one site (Table C in S2 Table).

## Discussion

### Edge effects on forest beetles

Our study found that the beetle communities of dry forest were affected by the creation of edges between remnant forest and open anthropogenic habitat. They were also influenced by spatial location (site) and local canopy cover, but not by other measured habitat variables. The only edge effect was at ground level, where species richness declined logarithmically with distance into the forest, together with altered species composition. This was due to a range of individual species whose edge responses were characterised by increased abundances near edges, with fewer species increasing in abundance away from edges. Total beetle abundance was unaffected by distance from the forest edge. However there was a greater rate of species accumulation per individual in edge compared with interior positions. This indicates that the edge-associated increase in total species per trap was not driven by any differences in numbers of sampled individuals.

Within the canopy, distance from the forest edge had no effects, and indicator analysis revealed no species-specific abundance increases associated with either edge or interior. Major et al. [31] likewise found a lack of distinct ‘‘interior” species among arboreal Coleoptera and Hemiptera in woodland remnants surrounded by anthropogenic pasture grassland in south-eastern Australia. The difference in edge responses between ground and canopy beetles is not surprising, for two reasons. First, insects inhabiting the canopy may react differently to the forest edge than those inhabiting the understory due to differing insect communities in each of these strata. Both this study and a range of previous studies across many forest types worldwide have found strong differences in beetle [32, 33] and arthropod [34] species composition between ground and canopy. Different types of response to forest edges may therefore be mediated by different ecological traits in ground and canopy, for example there is a larger proportion of poor flyers at ground level [34]. Second, the abiotic properties of micro-environments differ among vertical forest strata, for example the canopy level of moist tropical forest has greater wind velocity, more ultraviolet light and higher air temperature than the ground level [34]. Patterns of edge-interior difference in abiotic factors are also likely to differ among vertical strata, and this could drive contrasting community edge-responses. For example, abiotic conditions at the exposed forest edge may resemble those throughout forest canopies, whereas those on the ground in sheltered forest interiors may diverge [35].

Most previous studies of how forest beetle communities are affected by high-contrast edges with open anthropogenic habitats have sampled only at ground level (Table 2). Only one previous study [11] both compared beetle edge responses between ground and canopy and met the selection criteria of sufficient spatial replication and species-level identification. That study found similar results at ground and canopy levels, namely an increase in species richness, together with a change in species composition, within about 50 m of edges between European boreal forest and grassland, although the ground-level edge response was strongest.

**Table 2:** Effects of edges with open habitats on forest beetle communities globally.

											Community edge responses^7^:			Effect
Study no.	Region	Zone^2^	Matrix type^3^	Latitude	Area (km^2^)^4^	Ht	Trap type^5^	Max. dist (m)^6^	No of beetles	No. of species	Total abundance	Species richness	Species comp.	depth (m)
1^1^	Australia	ST	U	27°28’ S	380	G	FIT	256	3,605	578	N	I	Y	10
2 [36]	Europe	BO	U	60°17’ N	?	G	PT	60	4,301	40	-	-	Y	-
3 [11]	Europe	TE	O	51°5 N	80	G	FIT	500	13,204	536	-	I	Y	50
4 [37]	Europe	BO	U	50°89’ N	220	G	PT	100	52,198	99	-	-	N	-
5 [38]	Europe	BO	U	50°80’ N	240	G	PT	100	53,594	100	N	I	-	30
6 [39]	Europe	BO	O	50°05’ N	219,000	G	PT	100	17,723	162	N	I	Y	<50
7 [12]	Europe	TE	P	42°0’ N	40	G	PT	75	-	-	I	I	Y	15
8 [40]	Africa	TR	P & O	7°27’ N	5	G	PTB	160	7,705,	352	D	D	Y	10
9 [41]	Africa	TR	O	5°1 N	36	G	PT	420	12,582	22	D	N	Y	<30
10 [6]	S. America	TR	P	2°25’ S	1,100	G	LE	420	8,454	993	-	I	Y	13
11 [42]	Africa	ST	O	29°35’ S	5	G	PT	64	4,042	23	N	N	-	-
12 [10]	New Zealand	TE	P	42°32’ S	220	G	FIT, PT	1024	26,312	769	-	I	Y	4
13 [8]	New Zealand	TE	P	43°42’ S	420	G	FIT, PT	1024	35,461	893	N	I	Y	16
14 [7]	New Zealand	TE	P	43°44’ S	320	G	FIT, PT	46	6,586	283	N	N	Y	9
1^1^	Australia	ST	U	27°28’ S	380	C	FIT	256	3,605	578	N	N	N	-
3 [11]	Europe	TE	O	51°5 N	80	C	FIT	500	13,204	536	-	I	Y	3

Studies were obtained from a Web of Science search using terms: ‘beetles* AND edge effects’, ‘insects* AND edge effects’, ‘beetles* AND fragmentation’ and ‘insects* AND fragmentation’; but excluded if <6 transects perpendicular to a clear cut edge, or <3 sampling points per transect, or if not sorting to species, or if the matrix habitat was not anthropogenic or the edges lacked a clear forest to non-forest contrast. Ht is trap height: G ground, C canopy. In columns for beetle responses, I, D and Y indicate statistically significant edge effects ((P < 0.05); I = increase, D = decrease nearer to edges, N no significant effect, dashes where information was not provided or irrelevant.

^1^1 Current study.

^2^Latitudinal zones: TR tropical, ST subtropical, TE temperate, BO boreal

^3^Matrix habitat types: P mainly pasture, U mainly urban/suburban, O other (Study 3 a mix of grassland and regenerating woody vegetation; Study 6 an adjacent highway; Study 8 regenerating woody vegetation 3-years old; Study 9 sun grown coffee plantations; Study 11 grassland of unknown land use)

^4^Approximate area across which replicate edge sites were distributed;? if unclear in the publication.

^5^FIT flight intercept traps, PT pitfall traps, PTB baited pitfall traps, LE littler extraction (Winkler bags). Most studies targeted all captured beetles; studies 2, 7, 5 and 11 targeted Carabidae only; study 8 targeted Scarabaeidae only; study 6 targeted Scarabaeidae and Staphylininae only.

^6^Greatest sampled distance (m) from the edge.

^7^Inferred from clear patterns in the publication’s Tables or graphics in cases where: edge responses were curvilinear (studies 3, 13) or the statistical tests included matrix as well as forest sites (studies 8, 9, 10, 11, 13, 14); or a separate beetle-specific test result was not provided (study 7).

At ground level, among 11 studies in different forest regions worldwide that meet these same selection criteria (including the present study), eight reported increased beetle species richness within about 5–50 m into the forest from edges between remnant forest and open anthropogenic habitats, while two found no difference and one found a decrease (Table 2). Among a slightly different set of 11 relevant studies, all but one found a change in species composition. And among nine studies that assessed both, seven reported both an increase in richness and a change in composition–indicating that more species increase than decrease in abundance near edges (Table 2). Among eight studies that analysed for changes in total beetle abundance, six found no edge effect, one reported an increase, and one a decrease near edges. Each of these studies investigated a potential beetle community gradient from the edge to maximum sampling distances of 46 m to >1024 m into the forest (varying among studies), with most finding an edge effect that penetrated <20 m from the edge, many being <10 m (Table 2).

Ewers and Didham [43], in a global review, noted that invertebrate species richness typically correlates negatively with distance into habitat fragments, notwithstanding varied results of different studies, together with contrasting species-specific edge responses. This was also found in both our study and in most of the spatially well-replicated studies included in our review of edge responses within the Coleoptera (Table 2). Discussions about the causes and consequences of edge effects have frequently focused on forest-dependent species showing decreased abundances near edges [10, 44]. However, a better understanding of the underlying causal processes leading to positive edge responses is needed. There are several possible non-exclusive causes of the increased ground-level species richness within a few tens of metres from the edge. Three are discussed below: novel forest-floor edge microhabitats that favour some previously-rare species; abiotic conditions that favour canopy spill-over; and matrix spill-over from adjacent suburbs.

First, at positions just inside forest edges, ground-level microhabitats and resources may change in a variety of ways. For closed-canopy tropical rainforest, a common expectation is that such edges (like upper canopies) experience greater diurnal temperature ranges and lower humidity [13], and similar arguments have been applied to dense extra-tropical forest remnants [35]. In drier forest types, the open canopy enables greater penetration of sunlight and wind, possibly reducing edge-interior differences [45], although edge-associated changes in microclimate and vegetation to depths of 20–60 m in both forests with 50–95% canopy cover and savanna with 5–50% canopy cover were found in Brazil [46]. In open forests, local variation in canopy cover may be of greater importance in driving variation in habitat suitability for insect species than is the case for closed forests [47]. Indeed, our study found that local canopy cover was the strongest (and only) local habitat predictor of variation in beetle community attributes.

In our study, edge positions had a greater canopy cover than positions in the forest interior, broadly similar to the “edge sealing” response at edges of closed tropical forest [2, 13]. Our observed edge effects on beetles may therefore have been indirectly driven by the edge-related response of vegetation. However, in that case, the other habitat variables associated with increased canopy cover (ground-level grass cover, fine woody debris and bare ground) should have been correlated with beetle community attributes, and this was not so. Furthermore, there was little overlap between the specific indicator species of higher canopy cover and those of forest edges (Table B in S3 Table). Increased canopy cover at edges does not therefore appear to be the main driver of our edge effects, although it remains possible that the changes are driven by locally-altered values of other habitat conditions and resources (such as a different or more diverse plant species composition at edges).

Second, if abiotic similarity between the forest canopy and ground-level positions near edges (see above), could favour a “spill-over” of increased abundances of locally-occurring species that are otherwise found mainly in the forest canopy. This would cause greater similarity in species composition between ground and canopy at edge compared with interior positions. However this was not seen in the present study. Furthermore, none of the 24 specific indicator species of canopy-level sampling were also represented among the five indicator species of the ground-level edges, whereas the latter included four indicator species of ground-level sampling (Tables A and B in S3 Table). Therefore the data reject canopy spill-over as a causal mechanism for the edge effects in our study.

Third, forest edges may experience an influx of species from nearby matrix habitats [9]. The suburbs bordering this study’s forest sites were medium-density (allotment sizes typically around 0.1 ha), often with well-developed gardens of grass, trees and shrubs, including scattered remnant or planted eucalypt trees [48]. Suburbs closer to the central business district (CBD) tended to be older, more densely developed, and included fewer large allotments >0.1 ha or open grazing land. It proved to be logistically infeasible to sample the beetle communities in this urban matrix during this study, so the matrix spill-over hypothesis could not be a directly tested. However, we could show that increased distance from CBD was not significantly associated with decreasing ground-level compositional similarity between edge and interior, thus not providing clear support for such a spill-over.

It is possible that different processes may be operating in different places to produce net results in which more species respond positively than negatively to the forest edges. In the present study, only one species (Chry 8, Family Chrysomelidae) had a clear edge-avoidance response, and this species was not an indicator of sites located far from the CBD or of local habitats with higher or lower canopy cover. A functional understanding of edge responses in forest beetle communities will require further studies that look more closely at the relationships between changes in composition, species-specific abundance responses, and both biotic and abiotic habitat factors.

### Implications for conservation management

Open dry forests were once widespread but have experienced high recent deforestation both globally [17] and in Australia [49]. Our results indicate that many forest beetle species were tolerant to the consequent ecological changes near edges of habitat remnants, with some responding positively, and a minority being edge-sensitive. Retention of remnant forest patches is essential for biodiversity conservation. However, even though the urgency is clear, there has been ongoing and complex debate about which conservation approach is best. Conservation projects could be aided by any of the various conceptual frameworks associated with the vast number of studies of habitat fragmentation which have accumulated since the 1980s [43, 50, 51], but different frameworks may lead to incompatible manegement recommendations.

For example, if edge-sensitive responses are common within a particular biotic assemblage, larger conserved forest patches will be needed, but when such responses are uncommon (as in the present study) different priorities may drive conservation decisions. Furthermore, if effect depths are shallow (such as the 10 m observed here), conserved compact forest patches as small as 10 ha would be bordered by relatively narrow strips of unsuitable habitat. On the other hand, longer term changes in local habitat properties within such small fragments present a more insidious longer term threat [52] to a larger proportion of species in the forest beetle community. In the study region these changes are likely to include the cascading effects of weed invasion and altered fire regimes, which may be difficult to avoid given the urban context.

Additionally, we found that differences in species composition among sites as little as 10 km apart were much stronger than the edge effects. Strong spatial differences in beetle species composition among tropical rainforest fragments at a similar scale were reported by Grimbacher et al. [13]. However, studies of fragmentation and edge effects have often disregarded such differences as irrelevant background variation or have not used methods capable of its detection. Nevertheless, high between-site (beta) diversity presents a further imperative for conservation management, since species accumulation curves across multiple small widely scattered fragments can then more rapidly achieve higher species richness than accumulation curves from sampling within larger but more spatially homogeneous patches, as found by [53]. High beta-diversity also means that even small degraded fragments may contain unique subsets of species [51].

Although the among-site variation in our study was correlated with the urban-rural gradient, it was not driven by edge-associated species. Furthermore, the strong variation in species composition with CBD distance was not accompanied by a significant change in the compositional difference between edge and interior positions. This indicates that the apparent "CBD distance" effect is unlikely to be due to urbanisation, but is rather a response to some other underlying spatially correlated variable. Given a background of non-random habitat clearing [51], the intrinsic environmental characteristics of forest patches are likely to differ in locations close to, compared with further from, the CBD. We conclude that, in this heavily cleared and urbanising region, the retention of a regional mosaic of multiple widely-distributed forest remnants of different sizes is desirable to conserve beetle diversity. Sustaining habitat values of the smaller forest fragments will also depend on active management of their vegetation structure and composition, at both ground and canopy levels.

## Supporting information

S1 Fig The flight intercept traps (FIT’s). (TIF)

S2 Fig The effects of edge distance on total beetle abundance. (TIF)

S3 Fig Beetle species composition between ground and canopy levels from NMDS ordination. (TIF)

S4 Fig Beetle species composition among-site variation at ground level in NMDS ordination. (TIF)

S5 Fig Beetle species composition between edge and interior (ground) in NMDS ordination. (TIF)

S1 Table List of all families of beetles sampled. (DOCX)

S2 Table Effects of height, site and edge distance on beetle community attributes. (DOCX)

S3 Table IndVal’s of height, edge distance, canopy cover and site on the beetle community. (DOCX)

S4 Table Ground level abundances at each site. (DOCX)

S5 Table Relationships of distances from the forest edge with local habitat characteristics. (DOCX)
