# Supplementary figures and images for: Edge effects and beta diversity in ground and canopy beetle communities of fragmented subtropical forest
Source: PLoS One. 2018 Mar 1;13(3):e0193369. doi: 10.1371/journal.pone.0193369 (PMC5832255; doi:10.1371/journal.pone.0193369)

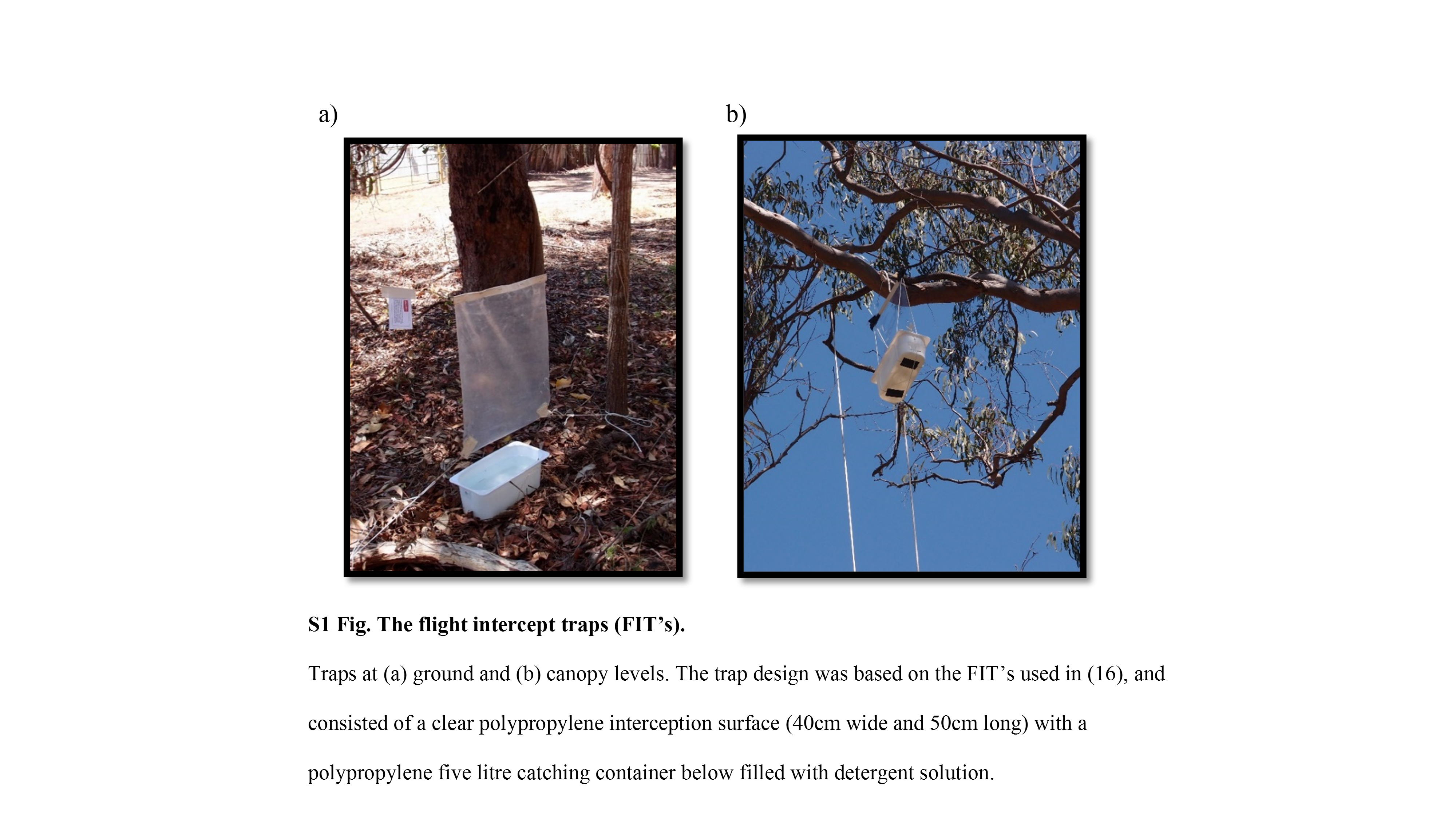

Supplement: S1 Fig — (TIF) [file pone.0193369.s001.tif]

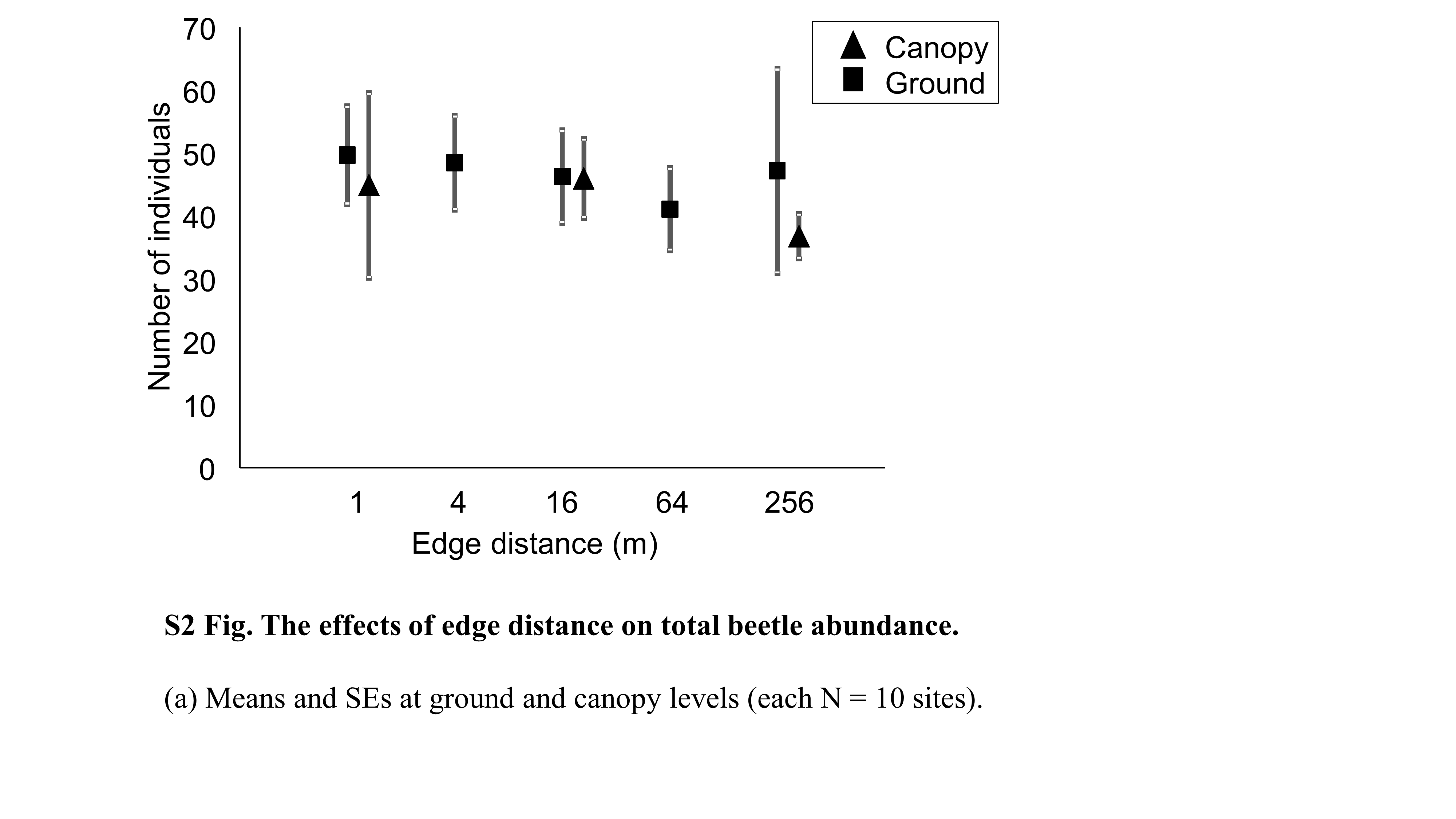

Supplement: S2 Fig — (TIF) [file pone.0193369.s002.tif]

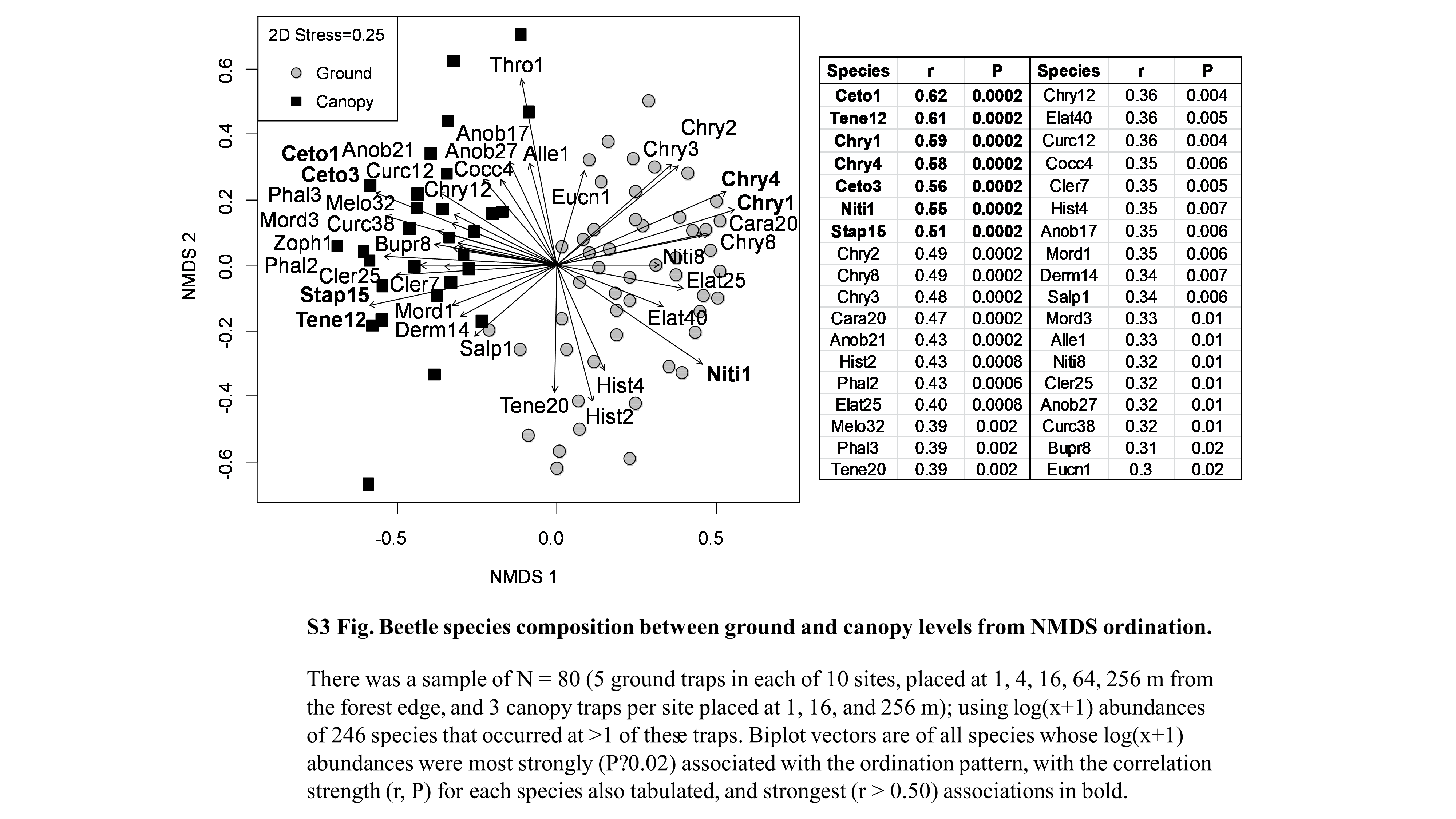

Supplement: S3 Fig — (TIF) [file pone.0193369.s003.tif]

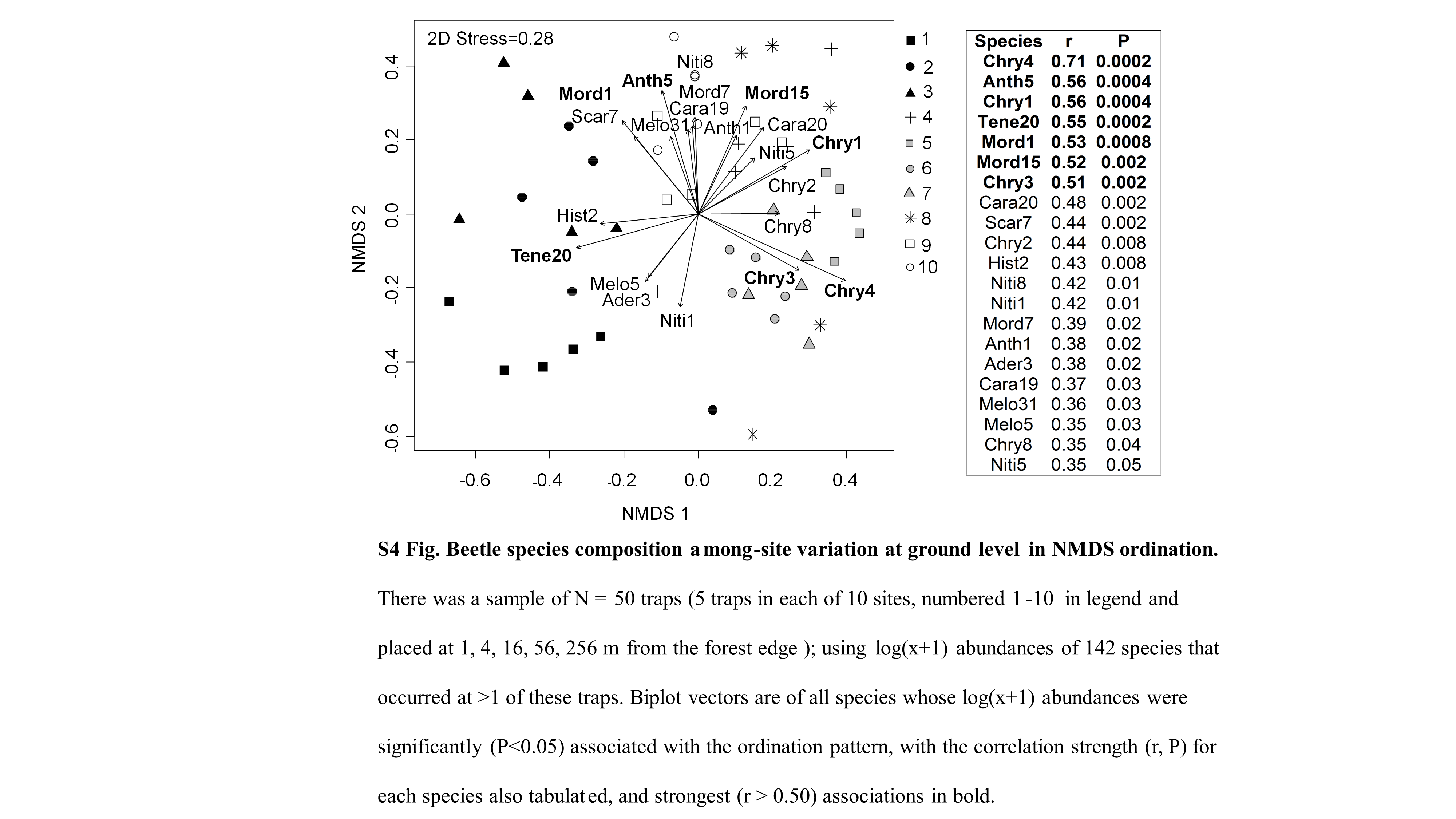

Supplement: S4 Fig — (TIF) [file pone.0193369.s004.tif]

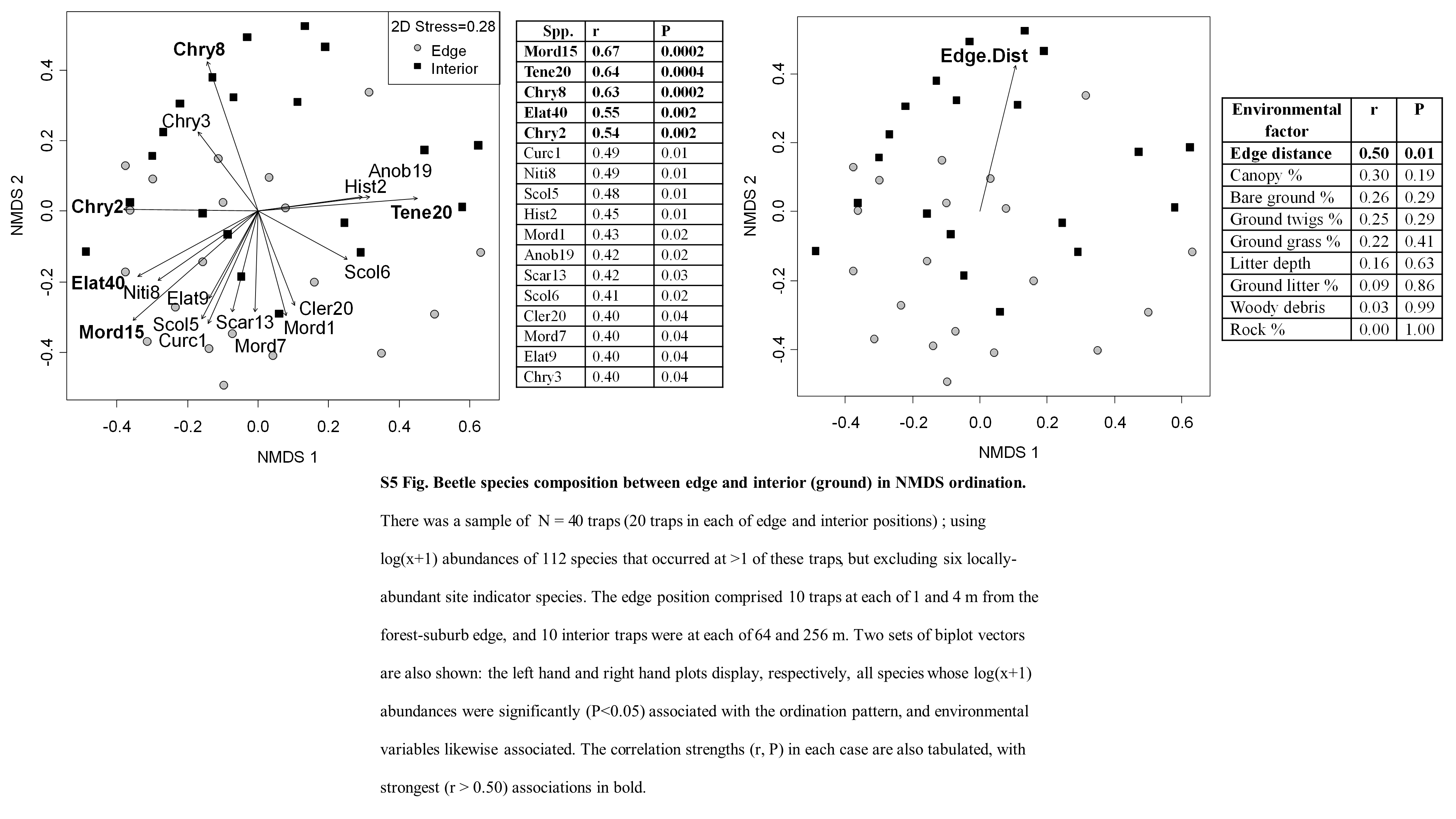

Supplement: S5 Fig — (TIF) [file pone.0193369.s005.tif]
