# Supplementary material for: Edge effects and beta diversity in ground and canopy beetle communities of fragmented subtropical forest
Source: PLoS One. 2018 Mar 1;13(3):e0193369. doi: 10.1371/journal.pone.0193369 (PMC5832255; doi:10.1371/journal.pone.0193369)
Supplement: S1 Table — (DOCX) [file pone.0193369.s006.docx]

**Supplementary material for:** Edge effects and beta diversity in ground and canopy beetle communities of fragmented subtropical forest.

Marisa J Stone, Carla P Catterall, and Nigel E Stork

**S1 Table. List of all families of beetles sampled.** List of all families, and subfamilies identified of beetles sampled using FITs at 10 sites in remnant dry forest patches of the greater Brisbane region (with 5 ground and 3 canopy traps per site; totalling 80 traps), in a four week period in November to December 2014, showing their numbers of species (Spp) and individuals (Indivs). The list is in taxonomic order.

| Family | Sub-family | Spp | Indivs | Family | Sub-family | Spp | Indivs |
| --- | --- | --- | --- | --- | --- | --- | --- |
| All Families | - | 578 | 3605 |  |  |  |  |
| Carabidae | - | 23 | 87 | Phalacridae | - | 9 | 44 |
| Histeridae | Chlamy-dopsinae | 4 | 28 | Erotylidae | - | 3 | 11 |
| Histeridae | Others | 3 | 4 | Coccinellidae | - | 21 | 50 |
| Leiodidae | - | 5 | 17 | Corylophidae | - | 5 | 6 |
| Scydmaenidae | - | 5 | 12 | Myceto-phagidae | - | 1 | 1 |
| Staphylinidae | Pselaphinae | 8 | 28 | Ciidae | - | 1 | 1 |
| Staphylinidae | Paederinae | 3 | 3 | Melandryidae | - | 2 | 3 |
| Staphylinidae | Scaphidiinae | 4 | 4 | Mordellidae | - | 16 | 512 |
| Staphylinidae | Others | 28 | 92 | Rhipiphoridae | - | 1 | 3 |
| Geotrupidae | - | 1 | 1 | Zopheridae | - | 6 | 55 |
| Scarabaeidae | Aphodiinae | 1 | 1 | Tenebrionidae | Lagriinae | 1 | 2 |
| Scarabaeidae | Scarabaeinae | 13 | 44 | Tenebrionidae | Alleculinae | 6 | 11 |
| Scarabaeidae | Dynastinae | 2 | 2 | Tenebrionidae | Others | 28 | 117 |
| Scarabaeidae | Melo-  lonthinae | 32 | 145 | Oedemeridae | - | 5 | 7 |
| Scarabaeidae | Rutelinae | 2 | 3 | Meloidae | - | 2 | 11 |
| Scarabaeidae | Cetoniinae | 3 | 71 | Salpingidae | - | 1 | 2 |
| Buprestidae | - | 23 | 53 | Anthicidae | - | 5 | 81 |
| Eucnemidae | - | 18 | 32 | Aderidae | - | 4 | 12 |
| Throscidae | - | 2 | 103 | Scraptiidae | - | 1 | 8 |
| Elateridae | - | 53 | 224 | Cerambycidae | - | 26 | 35 |
| Cantharidae | - | 1 | 1 | Chrysomelidae | Crypto-cephalinae | 8 | 20 |
| Dermestidae | - | 14 | 78 | Chrysomelidae | Eumolpinae | 5 | 61 |
| Anobiidae | Ptininae | 1 | 96 | Chrysomelidae | Others | 39 | 915 |
| Anobiidae | Others | 28 | 92 | Anthribidae | - | 5 | 10 |
| Cleridae | - | 36 | 91 | Belidae | - | 2 | 20 |
| Melyridae | - | 8 | 14 | Attelabidae | - | 1 | 2 |
| Nitidulidae | - | 10 | 215 | Brentidae | - | 3 | 5 |
| Silvanidae | - | 2 | 2 | Curculionidae | Scolytinae | 9 | 39 |
| Cucujidae | - | 1 | 1 | Curculionidae | Others | 40 | 113 |
| Laemophloeidae | - | 1 | 1 |  |  |  |  |
