# Supplementary material for: Edge effects and beta diversity in ground and canopy beetle communities of fragmented subtropical forest
Source: PLoS One. 2018 Mar 1;13(3):e0193369. doi: 10.1371/journal.pone.0193369 (PMC5832255; doi:10.1371/journal.pone.0193369)
Supplement: S2 Table — (DOCX) [file pone.0193369.s007.docx]

**Supplementary material for:** Edge effects and beta diversity in ground and canopy beetle communities of fragmented subtropical forest.

Marisa J Stone, Carla P Catterall, and Nigel E Stork

**S2 Table**. **Effects of height, site and edge distance on beetle community attributes.**

**S2(a)**. 1-factor ANOVAs (ground and canopy levels separately) of responses of total abundance and species richness to site and edge distance (both log-transformed). There were five edge distances on ground (1, 4, 16, 56, 256 m), three edge in canopy (1, 16, 256 m), 10 sites, and hence N = 10 traps per edge distance/height combination). Bolding indicates P<0.05.

| Factor | Height category | Dependent variable | F | df | P |
| --- | --- | --- | --- | --- | --- |
| Edge distance | Ground | Abundance | 0.36 | 4, 45 | 0.84 |
| Edge distance | Ground | Site-standardised abundance^1^ | 0.39 | 4, 45 | 0.82 |
| **Edge distance** | **Ground** | **Species richness** | **2.61** | **4, 45** | **0.048** |
| Edge distance | Canopy | Abundance | 0.02 | 2, 27 | 0.98 |
| Edge distance | Canopy | Species richness | 0.31 | 2, 27 | 0.73 |
| **Site** | **Ground** | **Abundance^2^** | **3.47** | **9, 40** | **0.003** |
| Site | Ground | Species richness | 0.93 | 9, 40 | 0.51 |
| Site | Canopy | Abundance | 0.70 | 9, 20 | 0.70 |
| Site | Canopy | Species richness | 0.74 | 9, 20 | 0.67 |

^1^Site standardised abundances at each trap were obtained by subtracting each site’s mean value from the sample value from each edge distance, to remove the site-specific variation.

^2^Tukey’s post-hoc tests revealed significant (P<0.05) differences between site 5 (mean = 91, SE= 24) and sites 1 and 2 (respective means 24, 30; SEs 4.6, 6.9)

**S2(b).** ANOSIMs beetle species composition in relation to height (canopy vs ground) edge distance (edge vs interior), 10 sites and five site-groups. Ground-level analyses of edge distance were repeated with exclusion of locally-abundant site indicator species. Species’ abundances were log(x+1) transformed. Bolding indicates P<0.05.

| Factor | Height category | No. of species | Excluded species | ANOSIM R | ANOSIM P | Total N (traps) |
| --- | --- | --- | --- | --- | --- | --- |
| **Height category** | Both | 246 | none | **0.64** | **0.0001** | 79^1^ |
| Edge category^2^ | Ground | 118 | none | 0.04 | 0.11 | 40 |
| **Edge category**^2^ | Ground | 106 | Site indicators^4^ | **0.06** | **0.04** | 40 |
| **Edge category**^2^ | Ground | 112 | Abundant^4^ site indicators | **0.07** | **0.03** | 40 |
| Edge category^3^ | Canopy | 109 | None | -0.05 | 0.78 | 20 |
| **Site (X 10)** | Ground | 142 | none | **0.60** | **0.0001** | 50 |
| **Site (X 10)** | Canopy | 109 | none | **0.32** | **0.001** | 29^1^ |
| **Site-group (X 5)** | Ground | 142 | none | **0.54** | **0.0001** | 50 |
| Site-group (X 5) | Canopy | 109 | none | 0.06 | 0.22 | 29^1^ |

^1^ The canopy trap at 16 m in site 10 had no beetles.

^2^ Edge vs interior (1 and 4 m vs 56 and 256 m from forest edge;10 ground traps per distance)

^3^ Edge vs interior (1 m vs 256 m from forest edge;10 canopy traps per distance)

^4^ Site indicators were identified using IndVal analyses; “abundant” indicators are those present in >20 (of 50) traps, but with disproportionately high abundance at certain sites; see Tables S3(b) and S4.

**S2(c).** Other tests of effects of edge distance and other environmental variables responses on beetle abundance (Abund), species richness (Rich) and species composition (Comp), on ground (N = 50 traps) and in canopy (N = 30 traps). Six “abundant” indicator species characteristic of particular sites were removed from all ground-level analyses of composition (these were present in >20 of 50 traps, but had disproportionately high abundance at certain sites; see Tables S3(b) and S4). Traps were replicates except for tests of CBD distance on abundance and richness where N = 10 sites. Total abundances and species richness values were log transformed; individual species abundances in composition analyses were log (x+1) transformed. Bolding indicates P<0.05.

| Environmental variable | Abund r^a^ | Abund P^a^ | Rich r^a^ | Rich P^a^ | Comp1 r^b^ | Comp1 P^b^ | Comp2 P^c^ |
| --- | --- | --- | --- | --- | --- | --- | --- |
| Spatial variables: |  |  |  |  |  |  |  |
| Ground level: |  |  |  |  |  |  |  |
| **Edge distance** | -0.17 | 0.24 | **-0.38** | **0.006** | **0.50** | **0.004** | 0.06 |
| **CBD distance** | **0.40** | **0.004** | 0.25 | 0.08 | **0.74** | **0.0002** | **0.0004**^d^ |
| Canopy level: |  |  |  |  |  |  |  |
| Edge distance | 0.02 | 0.92 | 0.08 | 0.67 | 0.22 | 0.52 | 0.60 |
| CBD distance | -0.03 | 0.87 | -0.05 | 0.79 | 0.24 | 0.45 | 0.15^d^ |
| Local habitat variables: |  |  |  |  |  |  |  |
| Ground level: |  |  |  |  |  |  |  |
| **Canopy %** | **0.30** | **0.03** | **0.36** | **0.01** | 0.30 | 0.19 | **0.03** |
| Woody debris | 0.11 | 0.45 | 0.00 | 0.99 | 0.03 | 0.99 | 0.78 |
| Litter depth | -0.02 | 0.89 | 0.25 | 0.08 | 0.16 | 0.61 | 0.52 |
| Ground litter % | 0.009 | 0.95 | 0.13 | 0.37 | 0.09 | 0.86 | 0.40 |
| Ground grass % | -0.09 | 0.53 | -0.23 | 0.11 | 0.21 | 0.41 | 0.28 |
| Ground twigs % | 0.18 | 0.21 | 0.15 | 0.30 | 0.06 | 0.31 | 0.12 |
| Bare ground % | 0.07 | 0.63 | 0.14 | 0.33 | 0.26 | 0.30 | 0.21 |
| Rock% | -0.12 | 0.41 | -0.05 | 0.73 | 0.00 | 1.00 | 0.31 |
| Canopy level: |  |  |  |  |  |  |  |
| **Canopy %** | -0.01 | 0.94 | -0.01 | 0.94 | **0.50** | **0.02** | 0.16 |
| Trap height | 0.24 | 0.20 | 0.25 | 0.20 | 0.07 | 0.93 | 0.16 |

^a^ Simple correlation analyses: Pearson’s r and P values with traps as replicates

^b^ Values of r and P from biplot vector overlays, using multiple correlation coefficients between values of environmental variables and scores on NMDS ordination axes 1 and 2; traps are replicates.

^c^ P values from DISTLM tests directly relating inter-trap Bray-Curtis dissimilarities to environmental variables, other than for inter-site and CBD distances (see d below); traps are replicates.

^d^ P values from Mantel tests with sites as replicates, comparing dissimilarities in species composition between site-pairs with their differences in distance from the central business district; at ground and canopy levels respectively the Mantel’s r values were 0.66 and 0.20.
