# Supplementary material for: Edge effects and beta diversity in ground and canopy beetle communities of fragmented subtropical forest
Source: PLoS One. 2018 Mar 1;13(3):e0193369. doi: 10.1371/journal.pone.0193369 (PMC5832255; doi:10.1371/journal.pone.0193369)
Supplement: S3 Table — (DOCX) [file pone.0193369.s008.docx]

**Supplementary material for:** Edge effects and beta diversity in ground and canopy beetle communities of fragmented subtropical forest.

Marisa J Stone, Carla P Catterall, and Nigel E Stork

**S3 Table. IndVal’s of height, edge distance, canopy cover and site on the beetle community.** Results of diagnostic indicator species analyses conducted after ANOSIM analyses showed significantly different species composition. IndVal and P show species’ indicator values and significance (Dufrene & Legendre 1997), based on log(x+1) abundance data. Species were included in a given analysis if present at 5 or more of the traps in the analysed set (Freq shows the number of traps where present). Mean abundances show the two values relevant to each indicator comparison.

**S3(a).** Canopy versus ground comparison, using 80 traps – 50 on ground and 30 in canopy, with 79 analysed species. All species for which P<0.05 are shown.

| Indicator comparison | Species | Indicator of: | Mean abundances | IndVal | P | Freq. |
| --- | --- | --- | --- | --- | --- | --- |
| Canopy vs ground |  |  |  |  |  |  |
|  | Tene12 | Canopy | 0; 0.21 | 0.52 | 0.0002 | 12 |
|  | Zoph1 | Canopy | 0.01; 0.23 | 0.50 | 0.0002 | 19 |
|  | Ceto1 | Canopy | 0; 0.29 | 0.48 | 0.0002 | 14 |
|  | Ceto3 | Canopy | 0.01; 0.15 | 0.43 | 0.0002 | 14 |
|  | Thro1 | Canopy | 0.12; 0.25 | 0.41 | 0.005 | 31 |
|  | Chry12 | Canopy | 0.02; 0.25 | 0.39 | 0.0004 | 18 |
|  | Phal2 | Canopy | 0.01; 0.15 | 0.36 | 0.0002 | 12 |
|  | Stap15 | Canopy | 0; 0.13 | 0.33 | 0.0002 | 11 |
|  | Melo32 | Canopy | 0.01; 0.15 | 0.31 | 0.0004 | 13 |
|  | Mord7 | Canopy | 0.05; 0.20 | 0.30 | 0.02 | 22 |
|  | Cler7 | Canopy | 0; 0.11 | 0.28 | 0.0006 | 8 |
|  | Anob27 | Canopy | 0; 0.10 | 0.26 | 0.0004 | 9 |
|  | Mord3 | Canopy | 0.02; 0.14 | 0.25 | 0.01 | 14 |
|  | Anob21 | Canopy | 0; 0.08 | 0.24 | 0.001 | 7 |
|  | Zoph2 | Canopy | 0; 0.08 | 0.24 | 0.0006 | 7 |
|  | Bupr8 | Canopy | 0.02; 0.09 | 0.24 | 0.01 | 13 |
|  | Curc38 | Canopy | 0.01; 0.08 | 0.22 | 0.006 | 8 |
|  | Phal3 | Canopy | 0; 0.07 | 0.21 | 0.0008 | 6 |
|  | Melo37 | Canopy | 0.01; 0.09 | 0.19 | 0.006 | 7 |
|  | Cler25 | Canopy | 0; 0.05 | 0.17 | 0.005 | 5 |
|  | Cler37 | Canopy | 0; 0.09 | 0.17 | 0.006 | 5 |
|  | Anob17 | Canopy | 0.01; 0.05 | 0.15 | 0.02 | 6 |
|  | Mord9 | Canopy | 0.01; 0.10 | 0.15 | 0.02 | 6 |
|  | Cocc4 | Canopy | 0.01; 0.06 | 0.15 | 0.02 | 6 |
|  | Chry1 | Ground | 0.29; 0 | 0.62 | 0.0002 | 18 |
|  | Niti1 | Ground | 0.22; 0.08 | 0.60 | 0.0002 | 41 |
|  | Chry8 | Ground | 0.21; 0.02 | 0.46 | 0.0002 | 24 |
|  | Chry4 | Ground | 0.37; 0 | 0.44 | 0.0002 | 22 |
|  | Cara20 | Ground | 0.15; 0 | 0.42 | 0.0004 | 21 |
|  | Chry2 | Ground | 0.19; 0 | 0.42 | 0.0002 | 20 |
|  | Chry3 | Ground | 0.12; 0 | 0.38 | 0.0002 | 19 |
|  | Ptin1 | Ground | 0.21; 0.11 | 0.38 | 0.02 | 34 |
|  | Anth5 | Ground | 0.15; 0.04 | 0.37 | 0.008 | 25 |
|  | Elat25 | Ground | 0.08; 0 | 0.34 | 0.001 | 17 |
|  | Niti8 | Ground | 0.07; 0 | 0.30 | 0.001 | 15 |
|  | Elat40 | Ground | 0.03; 0 | 0.22 | 0.01 | 11 |
|  | Psel1 | Ground | 0.08; 0.01 | 0.21 | 0.02 | 13 |
|  | Scol5 | Ground | 0.03; 0.01 | 0.19 | 0.046 | 12 |
|  | Elat38 | Ground | 0.02; 0 | 0.16 | 0.04 | 8 |
|  | Hist2 | Ground | 0.02; 0 | 0.16 | 0.047 | 8 |
|  | Hist4 | Ground | 0.02; 0 | 0.16 | 0.04 | 8 |

**S3(b)**. For ground traps only, comparisons of:

(1) edge vs interior, using 20 position-site combinations – 10 edge (averaged across traps at 1 and 4 m from edge within each site), 10 interior (averages of 64 and 256 m), with 41 analysed species;

(2) dense vs sparse canopy, using 35 traps - 17 traps with canopy cover 50-85%, and 18 traps with canopy cover 15-30%, with 34 analysed species; and

(3) among the 10 sites, using 50 traps - 5 in each site, with 41 analysed species.

All species for which P<0.01 are shown in each case. Indicator analyses were also conducted for canopy traps at edge vs interior (10 traps at 1 m, and 10 traps at 256 m), using 23 species, but these yielded no species with P<0.01.

| Indicator comparison | Species | Indicator of: | Mean abundances | IndVal | P | Freq. |
| --- | --- | --- | --- | --- | --- | --- |
| Edge vs interior: |  |  |  |  |  |  |
|  | Elat25 | Edge | 0.37; 0.11 | 0.35 | 0.059 | 9 |
|  | Scol5 | Edge | 0.26; 0.03 | 0.31 | 0.036 | 7 |
|  | Anob22 | Edge | 0.23; 0.06 | 0.30 | 0.046 | 7 |
|  | Elat38 | Edge | 0.27; 0.03 | 0.27 | 0.057 | 6 |
|  | Mord12 | Edge | 0.19; 0 | 0.25 | 0.042 | 5 |
|  | Chry8 | Interior | 0.32; 0.76 | 0.42 | 0.083 | 12 |
| Dense vs sparse canopy: |  |  |  |  |  |  |
|  | Mord15 | Dense | 0.45; 0.25 | 0.57 | 0.01 | 15 |
|  | Elat38 | Dense | 0.18; 0 | 0.41 | 0.003 | 7 |
|  | Melo29 | Dense | 0.12; 0 | 0.29 | 0.02 | 5 |
|  | Anob22 | Dense | 0.12; 0.02 | 0.25 | 0.08 | 5 |
|  | Niti1 | Sparse | 0.29; 0.15 | 0.53 | 0.07 | 16 |
|  | Tene20 | Sparse | 0.02; 0.15 | 0.30 | 0.05 | 6 |
| Among 10 sites: |  |  |  |  |  |  |
|  | Hist4 | Site 3 | 1.80; 0.22 | 0.28 | 0.04 | 8 |
|  | Elat38 | Site 4 | 1.68; 0.20 | 0.29 | 0.03 | 8 |
|  | Chry1 | Site 5 | 7.02; 1.97 | 0.28 | 0.001 | 31 |
|  | Chry4 | Site 5 | 6.79; 1.59 | 0.32 | 0.002 | 22 |
|  | Niti1 | Site 6 | 5.02; 1.77 | 0.24 | 0.003 | 36 |
|  | Cara7 | Site 7 | 1.08; 0.10 | 0.33 | 0.04 | 5 |
|  | Thro1 | Site 8 | 3.18; 0.36 | 0.50 | 0.0008 | 13 |
|  | Psel1 | Site 8 | 1.75; 0.27 | 0.34 | 0.03 | 12 |
|  | Anth5 | Site 10 | 3.56; 0.83 | 0.32 | 0.005 | 22 |
|  | Ptin1 | Site 10 | 3.41; 1.11 | 0.25 | 0.007 | 27 |
|  | Cara20 | Site 10 | 2.33; 0.63 | 0.29 | 0.005 | 21 |
|  | Cara19 | Site 10 | 1.68; 0.14 | 0.46 | 0.004 | 7 |
