# Supplementary material for: Edge effects and beta diversity in ground and canopy beetle communities of fragmented subtropical forest
Source: PLoS One. 2018 Mar 1;13(3):e0193369. doi: 10.1371/journal.pone.0193369 (PMC5832255; doi:10.1371/journal.pone.0193369)
Supplement: S4 Table — (DOCX) [file pone.0193369.s009.docx]

**Supplementary material for:** Edge effects and beta diversity in ground and canopy beetle communities of fragmented subtropical forest.

Marisa J Stone, Carla P Catterall, and Nigel E Stork

**S4 Table.** **Ground level abundances at each site.** Abundances are (averaged across 5 traps, at 1, 4, 16, 64, 256 m from the forest edge, using log(x+1) of individuals per trap). Species shown occurred at 5 or more traps; an additional 298 less common species were also recorded. Freq. is the number of traps (of 50). Bolded values show the most abundant species-site values (>2.0); bolded species are the site indicators from IndVal analyses (see Table S4), and those with asterisks were considered to be “abundant” site-associated species in analyses.

|  |  | Site: |  |  |  |  |  |  |  |  |  |
| --- | --- | --- | --- | --- | --- | --- | --- | --- | --- | --- | --- |
| Species | Freq. | 1 | 2 | 3 | 4 | 5 | 6 | 7 | 8 | 9 | 10 |
| **Niti1*** | 36 | **2.38** | **3.30** | 1.38 | **2.84** | 0.60 | **5.02** | **2.51** | 0.78 | 1.20 | 0.95 |
| Mord15 | 34 | 0.48 | 1.92 | 0.60 | **2.46** | **2.05** | 1.08 | 0.90 | 1.60 | 2.56 | **3.05** |
| **Chry1*** | 31 | 0.30 | 0.60 | 1.56 | **5.96** | **7.02** | 0.60 | 1.98 | 1.32 | **3.26** | **2.16** |
| Mord1 | 27 | 1.20 | 1.08 | **2.53** | 0.95 | 0.30 | 1.08 | 0.00 | 0.30 | 1.51 | **3.10** |
| **Ptin1*** | 27 | 0.95 | 0.30 | 0.95 | 0.48 | 0.90 | 1.60 | 1.51 | 0.30 | **3.02** | **3.41** |
| Chry8 | 25 | 0.00 | 0.60 | 0.48 | 1.30 | 1.30 | 1.81 | 1.78 | 0.90 | **2.53** | **2.33** |
| **Anth5*** | 22 | 0.00 | 1.38 | 1.98 | 0.00 | 0.30 | 0.60 | 0.60 | **2.58** | 0.00 | **3.56** |
| **Chry4*** | 22 | 0.00 | 0.00 | 0.00 | 0.90 | **6.79** | **6.31** | **5.24** | 1.00 | 0.60 | 0.30 |
| **Cara20*** | 21 | 0.00 | 0.00 | 0.00 | 0.30 | 1.20 | 0.60 | 1.08 | 0.48 | 1.98 | **2.33** |
| Chry2 | 21 | 0.00 | 0.00 | 0.30 | 0.95 | 1.78 | 1.91 | 0.60 | **2.78** | 0.90 | **2.28** |
| Chry3 | 19 | 0.30 | 0.00 | 0.30 | 0.60 | 1.38 | 1.38 | 1.92 | 1.56 | 0.30 | 0.30 |
| Derm14 | 17 | 1.68 | 0.30 | 0.78 | 0.30 | 0.78 | 0.60 | 0.60 | 0.00 | 0.78 | 0.30 |
| Elat25 | 17 | 0.30 | 1.26 | 0.00 | 0.60 | 1.51 | 0.78 | 0.60 | 0.60 | 0.30 | 0.30 |
| Niti8 | 15 | 0.00 | 0.60 | 0.00 | 1.30 | 0.00 | 0.30 | 0.00 | 0.60 | 1.48 | 1.20 |
| **Thro1** | 13 | 0.00 | 0.00 | 0.00 | 0.30 | 0.78 | 0.48 | 1.08 | **3.18** | 0.60 | 0.00 |
| **Psel1** | 12 | 0.30 | 0.00 | 0.00 | 0.00 | 0.00 | 0.60 | 0.00 | 1.75 | 0.60 | 0.90 |
| Curc1 | 11 | 0.48 | 1.38 | 0.00 | 1.38 | 0.60 | 0.60 | 1.08 | 0.00 | 0.00 | 0.00 |
| Elat40 | 11 | 0.00 | 1.38 | 0.00 | 1.00 | 0.60 | 0.00 | 0.48 | 0.00 | 0.60 | 0.30 |
| Scol5 | 11 | 0.60 | 0.60 | 0.30 | 0.78 | 0.00 | 0.00 | 0.30 | 0.00 | 0.30 | 0.90 |
| Mord7 | 10 | 0.00 | 0.60 | 0.30 | 0.48 | 0.48 | 0.30 | 0.00 | 0.00 | 0.30 | 1.38 |
| Anob22 | 9 | 0.00 | 1.08 | 0.30 | 0.60 | 0.30 | 0.30 | 0.00 | 0.30 | 0.00 | 0.30 |
| Anth1 | 9 | 0.00 | 0.00 | 0.30 | 0.30 | 0.00 | 0.30 | 0.60 | 1.08 | 0.30 | 0.00 |
| Beli1 | 8 | 0.00 | 0.00 | 0.30 | 0.48 | 0.30 | 0.60 | 0.00 | 0.60 | 0.60 | 0.00 |
| Elat22 | 8 | 0.30 | 0.30 | 0.00 | 0.78 | 0.00 | 0.00 | 0.00 | 0.30 | 0.60 | 0.60 |
| **Elat38** | 8 | 0.30 | 0.00 | 0.00 | 1.68 | 0.30 | 0.30 | 0.00 | 0.00 | 0.60 | 0.30 |
| Hist2 | 8 | 0.60 | 0.30 | 0.60 | 0.00 | 0.00 | 0.00 | 0.30 | 0.00 | 0.30 | 0.30 |
| **Hist4** | 8 | 0.48 | 0.60 | 1.80 | 0.00 | 0.60 | 0.30 | 0.00 | 0.00 | 0.00 | 0.00 |
| Mord2 | 8 | 0.00 | 0.00 | 0.60 | 0.00 | 0.30 | 0.30 | 0.30 | 0.00 | 0.00 | 1.08 |
| Tene20 | 8 | 1.20 | 0.48 | 1.38 | 0.00 | 0.00 | 0.00 | 0.30 | 0.00 | 0.00 | 0.00 |
| **Cara19** | 7 | 0.00 | 0.00 | 0.00 | 0.00 | 0.00 | 0.00 | 0.00 | 0.00 | 1.26 | 1.68 |
| Elat26 | 7 | 0.00 | 0.60 | 0.00 | 0.48 | 0.30 | 0.00 | 0.70 | 0.30 | 0.30 | 0.00 |
| Elat9 | 7 | 0.00 | 1.08 | 0.00 | 0.00 | 0.48 | 0.00 | 0.00 | 0.00 | 0.30 | 0.78 |
| Mord12 | 7 | 0.00 | 0.00 | 0.00 | 0.30 | 0.30 | 0.00 | 0.30 | 0.00 | 0.78 | 0.78 |
| Anob26 | 6 | 0.60 | 0.00 | 0.00 | 0.00 | 0.00 | 0.30 | 1.00 | 0.00 | 0.30 | 0.00 |
| Elat31 | 6 | 0.30 | 0.00 | 0.00 | 0.00 | 0.30 | 0.00 | 0.00 | 0.30 | 0.00 | 0.90 |
| Melo36 | 6 | 0.00 | 0.60 | 0.00 | 0.00 | 0.00 | 0.60 | 0.00 | 0.30 | 0.00 | 0.30 |
| Mord8 | 6 | 0.00 | 0.00 | 0.00 | 0.00 | 0.60 | 0.30 | 0.00 | 0.48 | 0.30 | 0.30 |
| Scar13 | 6 | 0.00 | 0.60 | 0.00 | 0.30 | 0.48 | 0.30 | 0.00 | 0.00 | 0.00 | 0.30 |
| Tene27 | 6 | 0.30 | 0.00 | 0.00 | 0.60 | 0.00 | 0.30 | 0.00 | 0.00 | 0.60 | 0.00 |
| Ader4 | 5 | 0.30 | 0.00 | 0.00 | 0.30 | 0.00 | 0.00 | 0.00 | 0.30 | 0.30 | 0.30 |
| Anob19 | 5 | 0.78 | 0.00 | 0.00 | 0.00 | 0.00 | 0.30 | 0.30 | 0.00 | 0.00 | 0.30 |
| **Cara7** | 5 | 0.00 | 0.00 | 0.00 | 0.00 | 0.30 | 0.00 | 1.08 | 0.60 | 0.00 | 0.00 |
| Chry12 | 5 | 0.00 | 0.00 | 0.00 | 0.78 | 0.00 | 0.00 | 0.00 | 0.30 | 0.48 | 0.30 |
| Chry9 | 5 | 0.00 | 0.00 | 0.00 | 0.30 | 1.00 | 0.30 | 0.30 | 0.00 | 0.00 | 0.30 |
| Elat4 | 5 | 0.00 | 0.00 | 0.00 | 0.30 | 0.00 | 0.00 | 0.00 | 0.60 | 0.30 | 0.30 |
| Melo29 | 5 | 0.30 | 0.00 | 0.30 | 0.00 | 0.00 | 0.00 | 0.00 | 0.30 | 0.30 | 0.60 |
| Mord3 | 5 | 0.48 | 0.30 | 0.30 | 0.00 | 0.30 | 0.00 | 0.00 | 0.00 | 0.00 | 0.48 |
| Niti5 | 5 | 0.00 | 0.00 | 0.00 | 0.00 | 0.00 | 0.00 | 0.30 | 0.60 | 0.60 | 0.00 |
| Scol8 | 5 | 0.00 | 0.00 | 0.78 | 0.00 | 0.60 | 0.60 | 0.00 | 0.00 | 0.30 | 0.00 |
