# Supplementary material for: Edge effects and beta diversity in ground and canopy beetle communities of fragmented subtropical forest
Source: PLoS One. 2018 Mar 1;13(3):e0193369. doi: 10.1371/journal.pone.0193369 (PMC5832255; doi:10.1371/journal.pone.0193369)
Supplement: S5 Table — (DOCX) [file pone.0193369.s010.docx]

**Supplementary material for:** Edge effects and beta diversity in ground and canopy beetle communities of fragmented subtropical forest.

Marisa J Stone, Carla P Catterall, and Nigel E Stork

**S5 Table. Relationships of distances from the forest edge with local habitat characteristics.**

**S5(a)** Correlations (Pearson’s r) between local habitat attributes and log(edge distance); N = 50 ground-level trap locations (10 sites, each with 5 edge distances). Bolding indicates P<0.05.

|  | **Canopy %** | Woody debris | Litter depth | Ground litter% | **Ground grass%** | **Ground twigs%** | **Bare ground%** | Rock% |
| --- | --- | --- | --- | --- | --- | --- | --- | --- |
| r | **-0.36** | -0.18 | -0.22 | -0.17 | **0.41** | **-0.43** | **-0.31** | -0.01 |
| P | **0.009** | 0.21 | 0.12 | 0.24 | **0.003** | **0.002** | **0.03** | 0.94 |

**S5(b)** Effect of edge distance on % canopy cover (mean and SE across n = 10 sites for each distance). ANOVA gave F = 2.47, df = 4, 45, P = 0.06.

| Edge distances: | 1 m | 4 m | 16 m | 64 m | 256 m |
| --- | --- | --- | --- | --- | --- |
| Mean | 52.0 | 49.0 | 35.5 | 32.5 | 35.5 |
| SE | 48.8 | 40.6 | 29.5 | 26.9 | 29.5 |
